# Supplementary material for: Genome-Wide Survey of the RWP-RK Gene Family in Cassava (Manihot esculenta Crantz) and Functional Analysis
Source: Int J Mol Sci. 2023 Aug 18;24(16):12925. doi: 10.3390/ijms241612925 (PMC10454212; doi:10.3390/ijms241612925)
Supplement: Supplementary file 1 [file ijms-24-12925-s001.zip › Supplementary Table S5. RWP-RK gene sequence of Arabidopsis thaliana, rice and cassava.pdf]

Supplementary data 1. RWP-RK protein sequences used for phylogenetics and motif analyses.

>AtNLP1

MEDDGGSDGGEGNGGFSPNSSFGAFADTAMDLDFMDELLFDGCWLETTDSKSLKQTEQSPSASTA
MNDNSPFLCFGENPSQDNFSNEETERMFPQAEKFLLEEAEVGKSWWIAPSASEGPSSSVKERLLQAIS
GLNEAVQDKDFLVQIWVPIQQEGKSFLTTWAQPHLFNQEYSSLAEYRHVSETYNFPADEGMKDFVG
LPGRVFLQKFPEWTPDVRFFRRDEYPRIKEAQKCDVRGSLALPVFERGSGTCLGVVEIVTTTQKMNYR
QELEKMCKALEAVDLRSSSNLNTPSSEFLQVYSDFYCAALPEIKDFLATICRSYDFPLALSWAPCARQG
KVGSRHSDENFSECVSTIDSACSVPDEQSKSFWEACSEHHLLQGEGIVGKAFEATKLFFVPEVATFSKT
NYPLAHHAKISGLHAALAVPLKSKSGLVEFVLEFFFPKACLDTEAQQEMLKSLCVTLQQDFRSSNLFIK
DLELEVVLPVRETMLFSENLLCGAETVESLTEIQMQESSWIAHMIKANEKGKDVSLSWEYQKEDPKELS
SGRENSQLDPVPNNVPLEAEQLQQASTPGLRVDIGPSTESASTGGGNMLSSRRPGEKKRAKTEKTIGL
EVLRQYFAGSLKDAAKSIGVCPTTLKRICRQHGIMRWPSRKIKKVGHSLKKLQLVMDSVQGAQGSIQL
DSFYTSFPELNSPNMSSNGPSLKSNEQPSHLNAQTDNGIMAEENPRSPSSSCSKSSGSSNNNENTG
NILVAEDADAVLKRAHSEAQLHNVNQEETKCLARTQSHKTFKEPLVLDNSSPLTGSSNTSLRARGAIK
VKATFGEARIRFTLLPSWGFAELKQEIARRFNIDDISWFDLKYLDDDKEWVLLTCEADLVECIDIYRLTQT
HTIKISLNEASQVKLSGSFGNTGLS

>AtNLP2

MEGGRGGGDGNFLPNSNFGVFSDSAMDMDFMDELLFDGCWLETTDGKSLKQTMGQQVSDSTTM
NDNNNNSYLYGYQYAENLSQDHISNEETGRKFPPIPPGFLKIEDLSNQVPFDQSAVMSSAQAEKFLLE
ESEGGRRYWIAPRTSQGPSSSVKERLVQAIEGLNEEVQDKDFLIQIWLPIQQEGKNFLTTSEQPHFFNP
KYSSLKRYRDVSVAYNFLADEDSKESVGLPGRVFLKKLPEWTPDVRFFRSEEYPRIKEAEQCDVRGSLAL
PVFERGSGTCLGVVEIVTTTQKMNYRPELDNICKALESVNLRSSRSLNPPSREFLQVYNEFYYAALPEVS
EFLTLVCRVYDLPLALTWAPCARQGKVGSRHSDENFSECVSTVDDACIVPDHQSRHFLEACSEHHLL
QGEGIVGKAFNATKLFFVPEVTTFSKTNYPLAHHAKISGLHAALAVPLKNKFNSSVEFVLEFFFPKACL
DTEAQQDMLKSLSATLQQDFRSLNLFIDKELELEVVFPVREEVVFAENPLINAGTGEDMKPLPLEEISQ
EDSSWISHMIKANEKGKGVSLSWEYQKEEPKEEFMLTSGWDNNQIGSGHNNFLSEAEQFQKVTNSG
LRIDMDPSFESASFGVGQTLLGSRRPGEKRRTKTEKTIGLEVLRQYFAGSLKDAAKSIGVCPTTLKRICR
QHGITRWPSRKIKKVGHSLKKLQLVIDSVQGVQGSIQLDSFYTSFPELSSPHMSGTGTSFKNPNAQT
ENGVSAQGTAAAPKSPPSSSCSHSSGSSTCCSTGANQSTNTGTTSNTVTTLMAENASAILKRARSEV
RLHTMNQDETKSLSRTLSHKTFSEHPLFENPPRLPENSSRKLKAGGASKVKATFGEAKVRFTLLPTWGF
RELQHEIARRFNIDNIAPFDLKYLDDDKEWVLLTCEADLEECIDIYRSSQSRTIKISVHEASQVKLGGSFG
SIGLGPSL

>AtNLP3

MVGPFKKILHAHNQRFPPSSSSSLDPVVDDSSRKQTRIILSYLLHLSLSLHITYSLCLLHFFFGSSNPFSP
MEESNNSAVVDFPDNFMDQLLWEECWEEEATQHDQALSSPSGLKERVACAMGHLQEVMGERELLI
QLWVPVETRSGRVLSTEEQPYSINTFSQSQSLALYRDASAGYSFAAEVGSEQLVGLPGRVFLRRMPE
WTPDVRFFRKEEYPRIGYARRYQVRATLALPLFQGTSGNCVAVMEMVTTHRNLEYASQLSTICHALEA
FDLRTSQTSIVPASLKVTSSSSSSSRTEVASILQGICSSHGLPLAVTWGHQDSSSCLSALISASYAADHG
SRCFLAACSEHHLLGGEGIAGRAFATKKQCFATDVAIFSKWSYPLSHYAKMFDLHAALAVPILTRGN
RTVQFVLELFFPRDCLDIQTHSLTLASQLKLRFQSSPHLMVDDNQIAEEVRDAATPPLTQEDPKGKQV
SFSFSSASSLENRKRKTKAEKDITLDTLRQHFAGSLKDAAKNIGVCPTTLKRICRQNGISRWPSRKIKKV
GHSLRKLQVVMDSVEGVQGSLHLASFYSSFPQLQSSSSSSFPFINPTQTVHVPPKSPPSSSGSQSSSGS
STCCSSEEQQLGGFQKPALSHPQLLTLSSMHEDQRPVRVTSSLPPLPSATTPRKAKDGMKVKAMFGD

SMLRMSLLPHSRLTDLRREIAKRFGMDDVLRSNFSLKYLDDDQEWVLLTCDADLEECIQVYKSSSLKET
IRILVHHPLSRPSFGS
>AtNLP4
MEDSFLQSENVVMDADFMDGLLLDGCWLETTDGSEFLNIAPSTSSVSPFDPTSFMWSPTQDTSALC
TSGVVSQMYGQDCVERSSLDEFQWNKRWWIGPGGGGSSVTERLVQAVEHIKDYTTARGSLIQLWV
PVNRGGKRVLTTKEQPFSHDPLCQRLANYREISVNYHFSAEQDDSKALAGLPGRVFLGKLPEWTPDV
RFFKSEEYPRVHHAQDCDVRGTLAIPVFEQGSKICLGVIEVVMTTEMVKLRPELESICRALQAVDLRSTE
LPIPPSLKGCDLSYKAALPEIRNLLRCACETHKLPLAQTWVSCQQQNKSGCRHNDENYIHCVSTIDDA
CYVGDPTVREFHEACSEHHLLKGQGVAGQAFLTNGPCFSSDVSNYKKSEYPLSHHANMYGLHGAVA
IRLRCIHTGSADFVLEFFLPKDCDDLEEQRKMLNALSTIMAHVPRSLRTVTDKELEEESEVIEREEIVTPKI
ENASELHGNSPWNASLEEIQRSNNTSNPQNLGLVFDGGDKPNDGFGLKRGFDYTMDSNVNESSTF
SSGGFSMMAEKKRTKADKTITLDVLRQYFAGSLKDAAKNIGVCPTTLKRICRQHGIQRWPSRKIKKVG
HSLQKIQRVIDSVQGVSGPLPIGSFYANFPNLVSQSQEPSQQAKTTPPPPPPVQLAKSPVSSYSHSSN
SSQCCSSETQLNSGATTDPPSTDVGGALKKTSSEIELQSSSLDETILTLSSLENIPQGTNLLSSQDDDFLR
IKVSYGEEKIRLRMRNSRRLRDLLWEIGKRFSIEDMSRYDLKYLDEDNEWVLLTCDEDVEECVDVCRTT
PSHTIKLLLQASSHHFPERSSATEYSLWH
>AtNLP5
MENNSLPMDPAMDSSFMDGLLLEGCWLETTDASEFLNFSPSTSVAPFDPSSFMWSPTQDTSNSLSQ
MYGQDCPERSSLEDQNQGRDLSTFNRRWWIGPSGHHGFSVMERLVQAVTHIKDFTSERGSLIQLW
VPVDRGGKRVLTTKEQPFSHDPMCQRLAHYREISENYQFSTEQEDSDSSSRDLVGLPGRVFLGKVPE
WTPDVRFFKNEEYPRVQHAQDCDVRGTLAIPVFEQGSQICLGVIEVVMTTQMVKLSPDLESICRALQ
AVDLRSTEIPIPPSLKGPDFSYQAALPEIRNLLRCACETHKLPLAQTWVSCLKQSKTGCRHNDENYIHC
VSTIDDACYVGDPTVREFHEACSEHHLLKGQGVVGEAFLTNGPCFSSDVSSYKKSEYPLSHHATMFGL
HGTVAIRLRCIHTGSVDFVLEFFLPKNCRDIEEQRKMLNALSTIMAHVPRSLRTVTQKELEEEGDSMVS
EVIEKGVTLPKIENTTEVHQSISTPQNVGLVFDGGTTEMGELGSEYGKGVSVNENNTFSSASGFNRVTE
KKRTKAEKNITLDVLRQYFAGSLKDAAKSIGVCPTTLKRICRQHGIQRWPSRKIKKVGHSLQKIQRVIDS
VEGVSGHHLPIGSFYASFPNLAASPEASSLQQQSKITTFLSYSHSPPAKSPGSSCSHSSSCSSETQVIKE
DPTDKTRLVSRSFKETQTTHLSPSSQEDDFLRVKVSYEEEKIRFKMRNSHRLKDLLWEIAKRFSIEDV
SRYDLKYLDEDNEWVLLRCDDDVEECVDVCRSFPGQTIKLLLQLSSSYLPERSSVSGCLS
>AtNLP6
MELDDLDLSGSWPLDQITFASNFKSPVIFSSSEQPFSPLWSFSETSGDVGGELYSAAVAPTRFTDYSV
LLASSESETTTKENNQVPSPSWGIMPLENPDSYCAIKAKMTQALRYFKESTGQQHVLAQVWAPVKN
RGRYVLTTSGQPFVLGPNSNGLNQYRMVSLTYMFSLDGERDGELGLPGRVFRKKLPEWTPNVQYYSS
KEFSRLGHALHYNVQGTLALPVFEPSRQLCVGVVELIMTSPKINYAPEVEKVCKALEAVNLKTSEILNHE
TTQICNEGRQNALAEILEILTVVCETYKLPLAQTWVPCRHRSVLAFGGGFKKSCSSFDGSCMGKVCMS
TSDLAVYVVDAHVWGFRDACAEHHLQKGQGVAGRAFQSGNLCFCRDVTRFCKTDYPLVHYARMF
KLTSCFAVCLKSTYTGDDEYVLEFFLPPAITDKSEQDCLLGSLLQTMKQHYSSLKVVSETELCENNMSLE
VVEASEDGMVYSKLEPIRIHHPAQISKDYLELNAPEQKVSLNSDFMENNEVDDGVERFQTLDPIPEAK
TVKKSERKRGKTEKTISLEVLQQYFAGSLKDAAKSLGVCPTTMKRICRQHGISRWPSRKINKVNRSLTRL
KHVIDSVQGADGSLNLTSLSPRPWPHQIPPIDIQLAKNCPPTSTSPLSNLQDVKIENRDAEDSAGSSTS
RASCKVNPICETRFRLPTHNQEPSRQVALDDSDSSSKNMTNFWAHLTCQDTASPTILQHKLVSIKATY
REDIIRFKISPESVSITELKQQVAKRLKLETAAFELKYLDDDREWVSVSCDADLSECLDTSAAKANTL
RLSVHDVTFNFGSSCESSEETMMCL
>AtNLP7

MCEPDDNSARNGVTTQPSRSRELLMDVDDLDLDGSWPLDQIPYLSSSNRMISPIFVSSSSEQPCSPL
WAFSDGGGNGFHHATSGGDDEKISSVSGVPSFRLAEYPLFLPYSSPSAAENTTEKHNSFQFPSPLMSL
VPPENTDNYCVIKERMTQALRYFKESTEQHVLAQVWAPVRKNGRDLLTTLGQPFVLNPNGNGLNQ
YRMISLTYMFSVDSESDVELGLPGRVFRQKLPEWTPNVQYYSSKEFSRLDHALHYNVRGTLALPVFNP
SGQSCIGVVELIMTSEKIHYAPEVDKVCKALEAVNLKSSEILDHQTTQICNESRQNALAEILEVLTVVCE
THNLPLAQTWVPCQHGSVLANGGGLKKNCTSFDGSCMGQICMSTTDMACYVVDAHVWGFRDAC
LEHHLQKGQGVAGRAFLNGGSCFCRDITKFCKTQYPLVHYALMFKLTTCFAISLQSSYTGDDSYILEFF
LPSSITDDQEQDLLLGSILVTMKEHFQSLRVASGVDFGEDDDKLSFEIIQALPDKKVHSKIESIRVPFSGF
KSNATETMLIPQPVVQSSDPVNEKINVATVNGVVKEKKKTEKKRGKTEKTISLDVLQQYFTGSLKDAAK
SLGVCPTTMKRICRQHGISRWPSRKIKKVNRSITKLKRVIESVQGTDGGLDLTSMAVSSIPWTHGQTSA
QPLNSPNGSKPPELPNTNNSPNHWSSDHSPNEPNGSPELPPSNGHKRSRTVDESAGTPTSHGSCDG
NQLDEPKVPNQDPLFTVGGSPGLLFPPYSRDHDVSAASFAMPNRLLGSIDHFRGMLIEDAGSSKDLR
NLCPTAAFDDKFQDTNWMNNDNNSNNNLYAPPKEEAIANVACEPSGSEMRTVTIKASYKDDIIRFRI
SSGSGIMELKDEVAKRLKVDAGTFDIKYLDDDNEWVLIACDADLQECLEIPRSSRTKIVRLLVHDVTTN
LGSSCESTGEL
>AtNLP8
MENPFASREKGFGNYSDFPTEQMDGLSSNFGSGVRNLISDDMFNPSSELMNFDSLAAWCNSPSAT
DILFAQYGLSNSQPMPFGAFTSFHVADPKATSLTRSFYDLESSYYGEERSSAQEMNSQFHRSSDSDEL
SGKRRKVVNQKIGFPNVLNCTIPRSLSHSLDEKMLKALSLFMESSGSGEGILAQVWTPIKTGDQYLLST
CDQAYLLDPRFSQYREVSRRFTFAAEANQCSFPGLPGRVFISGVPEWTSNVMYYKTDEYLRMKHAID
NEVRGSIAIPILEASGTSCCAVMELVTSKEKPNFDMEMDSVCRALQAVNLRTAAIPRPQYLSSSQRDA
LAEIQDVLRTVCHAHKLPLALAWIPCRKDQSIRVSGQKSGENCILCIEETACYVNDMEMEGFVHACLE
HCLREKEGIVGKAFISNQPFFSSDVKAYDISEYPIVQHARKYGLNAAVAIKLRSTYTGEDDYILELFLPVS
MKGSLEQQLLLDSLSGTMQRICRTLRTVSEVGSTKKEGTKPGFRSSDMSNFPQTTSSENFQTISLDSE
FNSTRSMFSGMSSDKENSITVSQGTLEQDVSKARTPEKKKSTTEKNVSLSALQQHFSGSLKDAAKSLG
GETSAYFQAWVYFFCPTTLKRICRQHGIMRWPSRKINKVNRSLRKIQTVLDSVQGVEGGLKFDSATGE
FIAVRPFIQEIDTQKGLSSLDNDAHARRSQEDMPDDTSFKLQEAKSVDNAIKLEEDTTMNQARPGSF
MEVNASGQPWAWMAKESGLNGSEGIKSVCNLSSVEISDGMDPTIRCSGSIVEPNQSMSCSISDSSN
GSGAVLRGSSSTSMEDWNQMRTHNSNSSESGSTTLIVKASYREDTVRFKFEPSVGCPQLYKEVGKRF
KLQDGSFQLKYLDDEEEWVMLVTDSDLQECLEILHGMGKHSVKFLVRDLSAPLGSSGGSNGYLGTGL
>AtNLP9
MENPSASRDNKGFCFPDIPVEEMDGWVKNLISEEDMFSSSSTSELMNFESFASWCNSPSAADILFTQ
YGLSTSQSIIPFGGLEGSYACEKRPLDCTSVPRSLSHSLDEKMLKALSLFMEFSGEGILAQFWTPIKTGD
QYMLSTCDQAYLLDSRLSGYREASRRFTFSAEANQCSYPGLPGRVFISGVPEWTSNVMYYKTAEYLR
MKHALDNEVRGSIAIPVLEASGSSCCAVLELVTCREKPNFDVEMNSVCRALQAVNLQTSTIPRRQYLS
SNQKEALAEIRDVLRAVCYAHRLPLALAWIPCSYSKGANDELVKVYGKNSKECSLLCIEETSCYVNDM
EMEGFVNACLEHYLREGQGIVGKALISNKPSFSSDVKTFDICEYPLVQHARKFGLNAAVATKLRSTFT
GDNDYILEFFLPVSMKGSSEQQLLLDSLSGTMQRLCRTLKTVSDAESIDGTEFGSRSVEMTNLPQATV
SVGSFHTTFLDTDVNSTRSTFSNISSNKRNEMAGSQGTLQQEISGARRLEKKKSSTEKNVSLNVLQQY
FSGSLKDAAKSLGVCPTTLKRICRQHGIMRWPSRKINKVNRSLRKIQTVLDSVQGVEGGLKFDSVTGE
FVAVGPFIQEFGTQKSLSSHDEDALARSQGDMDEDVSVEPLEVKSHDGGGVKLEEDVETNHQAGPG
SLKKPWTWISKQSGLIYSDDTDIGKRSEEVNKDKEDLCVRRCLSSVALAGDGMNTRIERGNGTVEPN
HSISSSMSDSSNSSGAVLLGSSSASLEQNWNQIRTHNNSGESGSSSTLTVKATYREDTVRFKLDPYVV
GCSQLYREVAKRFKLQEGAFQLKYLDDEEEWVMLVTDSDLHECFEILNGMRKHTVKFLVRDIPNTAM

GSSAGSNGYLGTGT
>AtRKD1
MKSFCKLEYDQVFGKENNSFSFLNHSSLYSHQSELANPFFELEDEMLPSATSSNCFTSASSFLALPDLE
PISIVSHEADILSVYGSASWTAEETMFVSDFAKKSETTTTKKRRCREECFSSCSVSKTLSKETISLYFYMPIT
QAARELNIGLTLLKKRCRELGIKRWPHRKLMSLQKLISNVKELEKMEGEENEDKLRNALEKL
EKEKKTIEKLPDLKFEDKTKRLRQACFKANHKRKRRSGMSTPITSSSSSASASSSSYSSVSGFER
>AtRKD2
MADHTTKEQKSFSFLAHSPSFDHSSLSYPLFDWEEDLLALQENSGSQAFPFTTTSLPLPDLEPLSEDVL
NSYSSASWNETEQNRGDGASSEKKRENGTVKETTKKRKINERHREHSVRIISDITTYTTSSAPTTLSKET
VSRYFYMPITQAAIALNVGLTLLKRRCRELGIRRWPHRKLMSLNTLISNVKELQKMEGEENAEKLQDAL
EMLEKEKRTIEDLPDLEFKDKTKRLRQACFKANHKRKKKRSLKSDQSQVPSCSSSGSVPSDESV
DEAGMESDEEMKYLLCGFSSEFTSGL
>AtRKD3
MADQRPLMTWLEANNYESFLQEDIFSFLDQSLFVDPHSSFIDPFKDFQTQNWFSLQDSIVNHISTTFA
ADHTFLASLDLEAISSTFSLDISSGWWNENNGNYNNQVEPNLDEISRTNTMGDPNMEQILHEDVNT
MKEKTSQKRIIMKRRYREDGVINNMSREMMKQYFYMPITKAAKELNIGVTLLKKRCRELGIPRWPHRK
LTSLNALIANLKDLLGNTKGRTPKSKLRNALELLEMEKKMIEEVPDLEFGDKTKRLRQACFKAKYKRRRL
FSSSS
>AtRKD4
MSSSKHSSVFNYSALFLSLFLQQMDQNSLHHLDSPKIENEYEPDSLYDMLDKLPPLDSLLDMEDLKPN
AGLHFQFHYNSFEDFFENIEVDNTIPSDIHLLTQEPYFSSDSSSSSPLAIQNDGLISNVKVEKVTVKKKR
NLKKKRQDKLEMSEIKQFFDRPIMKAAKELNVGLTVLKKRCRELGIYRWPHRKLKSLNSLIKNLKNVGM
EEEVKNLEEHRFLIEQEPDAELSDGTKKLRQACFKANYKRRKSLGDDYY
>AtRKD5
MVDQGFFTLKKEKKKNILIKLFYVKILGFCFCDSWSSSDMAHSLTSLAVFQSVIRKEMVRSLHVYESV
EIEREFWFKSKSCYVEKKAKPLFRSEDFRRPEISEGSVFGTWRCIFVFRFNHSLPRFPTLLCLSRNPK
LEDIPNLANELKFISELKPSKIYEEEQCSSSTEGYYNSDLPKPRKLVLKQDLNCLPDSETESEESVNE
KTEHSEFENDKTEQSESDAKTEILKKKKRTPSRHVAELSLEELSKYFDLTIVEASRNLKVGLTVLKKK
CREFGIPRWPHRKIKSLDCLIHDLQREAEKQQEKNEAAAMAVAKKQEKLETEKRNIVKRPFMEIGIET
KKFRQENFKKRHRASRAKKNQESLVTSSST
>AtRWP-RK-1
MAGDDPKSSAPKDDGYEDPYINDPFDGFGPFTDEDLINVMADINPNLMDMSFTEHTPPSVLPTLPSD
VDETHLFHELDLSISNYDSKDEFETLSFVEDGFTIPLDMFEQPNDEHNDNNDVPGEIGNDNGKNVVG
DSTRLELSTRVFGGANGLDFEIGGSATMPVPVTTTISDGVHACNCCRLLRELVHLKEGEERSKLDIYGGI
GFICHAILLIQLLAPDSMQRQPLIFHLQHLTMEEVKKFIEDYCYQRVANGLSLLQDTNAAFYQAMSAN
SIFNQPPPMLTLPSSTDVPLSLLSPNEDHDVPLSLLSPEEALDIQPVPPHVELREEPTTRLPKQREKRQTP
LAAQVISLVSQFNI

>OsNLP1* in 2005 (OsNLP2)
MEQKPSPPPPPRSDEEEDGLMGCGMGGTGDIAGGDLDLMEEFLLATPGFDLSEFWHPGAASPFSPLF
DIGSSVTTLTTPAPAAGEDDRDEAEMPSRGGGGLEVSPAHRGWTFQTAPQEVAVEPTVKERLRRALE
RIASQSQSQAQRGDGELLVQVWVPTRIGDRQVLTTCGQPFWLDRRNQRLANYRTVSMKYQFSADE
SARADLGLPGRVFVGRVPEWTPDVRYFSTEEYPRVQHAQYFDIRGSVALPVFEPRSRACLGVVELVMT
TQKVNYSAEIENICNALKEVDLRSSDVSSDPRSKVVDASYRAIIPEIMDVLRAVCDTHNLPLAQTWIPCI

CQAKRGSRHSDESYKHCVSTVDEACYVRDCSVLGFHQACSEHHLFRGEGVVGRAFGTNEPCFSPDIT
TYSKTQYPLSHHAKLFGLRAAVAIQLRSVKTGSLDFVLEFFLPMKCINTEEQRAMLNSLSNTIQQVCYT
LRVVKPKELVNDGPFEISQPTRPEFYAKSVHEDLDELCSGINVPGRTTSLEASEEVSSWIASLVDAQNK
GGKGEIDVDLPFGFSKQDDEGFSVTAGWHTSPVMAPDGSMFSGFKRHEDYDVKENTCSSDPSNSN
SDKAVEKRRTKTEKTVSLQDLRKHFAGSLKEAAKNLGVCPTTLKRICRQHGINRWPSRKIKKVGHSLKK
LQMVIDSVHGPEGTVQLSSLYENFTKTTWSERELQGDVHFPASEQNFQLEPSVPDRPCEGRFTSHTS
GSNSISPSCSQSSNSSLGCSSVPKTQQQHGSAPQLAVKEEISMDENQCSTLIKSASHAEAELQMFVEE
RPTMLFRSQSQVLLSEHKPIENMSNVQKARSDSLKIKAIYGEERCIFRLQPSWGFQRLKEEIVKRFGISQ
DTHVDLKYLDDESEWVLLTCDADLLECIDVYKSSSNQTVRILVNPSIQPLLNASFGQTGLS

>OsNLP2* in 2005 (OsNLP6)

MDMPTPSNRAGCNGNTGGTMGPSDDPYGAAAMNLDCYSEIYSPSVADQLFSLLNDPAAHRMFA
MWPSMGSSPCAAGTSEDMPLDAYSGLGEAVEEPSQIMSVNPTEAEKTGKSSGELGSDDGAHQGSS
MVPRSVVGSSLADRMLMALSLFRESLGSGALAQVWMPVEQEGHVVLSTCEQPFLLDQVLAGYREVS
RHFVFSAKEEPGLQPGLPGRVFISGVPEWTSSVLYYNRPEYLRMEHALHHEIRGSLAMPIYDPSKDSCC
AVFELVTRKEKPDFSAEMDNVCNALQAVNLKATKGSSNQKFYTENQKFAFTEILDVLRAICHAHMLPL
ALTWVPTSNGIDGGYVVGKDGASFSQSGKTIIRIHESACYVNDGKMQGFLQACARRHLEKGQGIAGR
ALKSNLPFFSPDIREYSIEDYPLAHHARKFSLHAAVAIRLRSTYTGNDDYILEFFLPVSCKGSGEQQMLL
NNLSSTMQRICKSLRTVYEAEVDNVNAGTAAVFRKNNESCLPTGHTESSSHGDQSITGASFEDTSLA
NKPGVMEPELAEQVQPSSIGHAEKKRSTAEKNISLDVLRKYFSGSLKDAAKSLGVCPTTLKRICRHHGIS
RWPSRKINKVNRSLKKIQTVINSVHGVDRSLQYDPATGSLVPVVSLPEKLTFPSCDGLPTPSVGKTVEE
NSDLKSEEGCSLPDGSQRQSCQLQISDVKKSNEDEFHIGSGNSDFYGANATAKSNSEVTQGPLCPTG
AFSALHLKGTDCTNPSSSLRPSSESTRNQIVGRNSPSIQQEDLDMLDNHEAEDKDHMHPSTSGMTD
SSSGSASSHPTFKQNTRSALKDAASPALTVKATYNGDTVRFKFLPSMGWYHLLEEIAKRFKLPTGAYQ
LKYKDDEDEWVILANDSDLQECVDVLDSIGSRIVKLQVRDLPCIVSSSGSSTCLQLAAHSS

> OsNLP3* in 2005 (OsNLP1)

MEVDPSSSLPGAGEGGGGGIGGGGGDLWPFDSLTTSLLFSSVSASPQPLPASSSSWLTPPSPLWLFDE
RQLLPLDMGAPAAPATAPPAEAAAVVEEVHRTRSGNSDTTSKRVDQINSKWQFHLSIDDNTDSSCLF
KERLTQALRYFKESTDQHLLVQVWAPVKSGDRYVLTTSGQPFVLDQQSIGLLQYRAVSMMYMFSVD
GENAGELGLPGRVYKQKVPEWTPNVQYYSSTEYPRLNHAISYNVHGTVALPVFDPSVQNCIAVVELI
MTSKKINYAGEVDKVCKALEAVNLKSTEILDHPNVQICNEGRQSALVEILEILTVVCEEHKLPLAQTWV
PCKYRSVLAHGGGVKKSCLSFDGSCMGEVCMSTSDVAFHVIDAHMWGFRDACVEHHLQKGQGVS
GKAFIYRRPCFSKDISQFCKLEYPLVHYARMFGLAGCFAICLQSMYTGDDDYILEFFLPPNCRNEDDQ
NALLESILARMKKCLRTLKVVGNGDTNEVCLQISNVLIIETEDLKTNVHFENSEGCFRESPESNGSQRV
HEVDNDGNKVSIMSERHLLADDNSQNNGASVGRPNGSGASDSLHKSNKPPERRRGKAEKTISLDVL
QQYFSGSLKNAAKSLGVCPTTMKRICRQHGISRWPSRKINKVNRSLSKLKQVIESVQGSDAAFNLTSIT
GPLPIPVGPSSDSQNLEKASPNKVAELSNLAVEGDRDSSLQKPIENDNLAILMSQQGFIDANNNLQLE
ADKASHSRSSSGEGSINSRTSEASCHGSPANQTFVCKPIASTFAEPQLIPEAFTKEPFQEPALPLSRMLIE
DSGSSKDLKNLFTSAVDQPFLARSSNLALMQNSGTVTIKASFKEDIVRFRFPCSGSVTALKDEVAKRLR
MDVGMFDIKYLDDDHEWVKLACNADLEECMEISGSHVIRLLVSDVAAHLGSSCGSSG

>OsNLP4 (OsNLP4)

MEEGDPQPSISLARTPSEGAAAAVDLDLLEQLLSADNAWLEVAANTSRSPNFFATPSNCLTDASVAT
TTPANSWWIQPSGASTSVRERFDQALAYIRETQSDADVLVQLWVPVKGNDGQLVLTTSGQPFTLDQ
RSNSLIQFREVSTKYQFSADVASGSSPGLPGRVFIGRLPEWSPDVRYFTSYEYPRINHAQYLDVHGTM
GLPVFERGNYSCLGVIELIMTKQKLNFTSELNTICSALQAVNLTSTEVSSIPRAKLNSASYKDALPEILEV

LRAACITHKLPLAQTWVTCAQQGKRGSRHSDENYKYCISTIDAACYVNEPRMQSFHEACSEHHLLRG
QGVAGKAFTTNQPCFLPDIGSSTKLEYPLSHHAKIFNLKGAVAIRLRCTRTGIADFVLEFFLPTDCEVL
EEQKAVLDSLSGTMRSVCQTLRVVTDKEMEDEAMREMNELNSFSPRGKNKVEELSFGDNTRGDREE
ASWTTLVGTSQKGSDLAELHTHGMLSHGGHGSSQAGDQTSKEGSKVKRRTKTEKTVSLQVLRQYFA
GSLKDAAKSLGVCPTTLKRICRQHGINRWPSRKIKKVDHSLRKLQQIIDSVHGAETAFQLNTLYKDLTN
TSVSSDNNLSGSVTVPLANQNNLDFEMHQHHRLSSNIPSTSLSHSSCSQSSDSSPSCSGGATKHSPQ
VGADQVRSGCLPQHSPVQTLQTEAASINEHFSGQEAPIDLLQDVAEKANGEQHMSQSPSSPKQTA
NVGMRVKVTFGSEKVRFRLKPECDFQELKQEISKRLSIADMNSLIVKYLDDDSEWVLMTCDADLHECF
HVYKLADIQTIKISVHLAASPTTRITIGHTGFS
>OsNLP5 (OsNLP6)
MDMLMLSSALDGLDSYTDLVAGSSVADSIFSALTCFPPSQERLLHVSTPVGSNSRQDDSDVSITKEGT
TTRRGDCAAGLASGEPVAAGIGSVPKPFDGVTLTERMLRALAMLKEASGGEAILVQVWMPVRNGEQ
HVLTTSDQPFLLDQKLTGYREVSRQFTFSAEEGPGLFPGLPGRVFMSGMPEWTSNVMYYHGSEYLRV
DYARRHEVRGSLAMPVFNSSGGSCCAVLEVVMTREKDNFCLEMVNVSNALQSVQLSTVNAWRHS
QSYSRDQKLALMEIFDVLQAVCQAHLLPLALAWIPVCSKRDVLVSVEYGANFGKRNKEVLCIEESACY
VNDTRMRDFVQVCAEHPLEKGQGVAGNAYLSNNPFFSSDVKDYDMHAYPLVNHARKFGLHAAVA
IRLQSTYTKNDDYVLEFFLPVLCKGGGEQQLLLDSISATMRRVCKSLRTVSDAELKEDVTRKPSNENRS
GTRCPSPVNLIYSGREIDVSNETKTNTPLEYQIEGIDEQLSDTKSTNKLIKCSNASDGEKRRSSTEKSVSL
SVLQQYFSGSLKDAAKSIGVCPTTLKRICRQHGISRWPSRKIKKVNRSLKKIQNVISSVHGVEGVLKYDP
STGCLVSSVSPSIEPVLMNVEHKGSDPLPIESELPHLNFEPDCDAYRREHAGQDVLHKLQNKQNGEIN
FDMDDGELFRNSHSTRTLSGAFCEDMPNGLYVAREMTCVAKTGTRTERLEHKPSSRDSFSAPQEYR
MESETDKSNKNSKQSLPSSSSMTDCSTSSGETFKSIKSQSANESNKTVVVKASYKNDTIRFKLLPSMKY
EQLLEEIAKRLKLSIGLFQLKYKDDEGDWVILASDADLQECLEILDTTRLRILKLQVQDVVCPIGSSSGSC
SMLRP
>OsNLP6 (OsNLP7)
MERVVGDFNLLLQRGGAAGERGSEGGGGGSPGTEEAAAVAVKQRIARALRLYKEAAGDGGGGWM
VQVWAPARDGARRVLATRGQPFVLASQCHRLFQYRTVSLTRVFPVGGAAAADEQGLPARAFDATA
PEWTPNVQCYGSGEYARISYALIYDIQGSLALPILDPDDASSPLAVLELVTTAPLLRVSGEVANLCNALR
AVSLRGAGICNRAAECYSLAVNQIVHRDATRAAMAEVSELLITVCEAHKLPLAQTWVRCWSCGGGG
EDTEKAALTTAGAPFHLAAGADARGFRDACVEHHLQRGQGLVGTAARAPGGGRLCADVARCSKDD
YPLAHYAGMYGLAGCLVLRAELSAAAMADAAAATAGDEEDCVVLELFLPPDCTGVAEQKAAVDAVS
ATIKQCSGNLKAIVISNLDDLFLDTMADGDHQLRHEMDDLGDDQRCSDEEDLQLLENTNIGELNIHN
ADQIRNEDPTSQVGKNKTKRGKAEKSVTLEELQKHFSGSLKDAARSLGAGYSVDYLHQNPDVYAQIN
TQPLKVGSPGSMASQGGHFARSARCGNGDGVVTIKASHRGDIIRFRVPCSAGVAAVKAVVAKRLSL
DAGAFDVKYLDDDHEWVLLSCDADFQECLDVVPALPSMSVTARSGSGAAAPVVVRLMVQEVADNI
GSSCASSD
>OsRKD1 in 2005 (OsRWP-RK1)
MCAAPMASTAQPQQQPQQQQEQQPVAAAAVPTPAPPASEAQPQKPTRVSLSYEEISKLFSLPIAEA
ASILGVCTSVLKRICRSHGIVRWPYRKLVSGKSGDDTKNAEREKAKGLLEISKVAKQKALSASGLSTVSP
GAFQGVAKSQQGSSKAGQVSPPGKQNVLGGSAILSYGTQTKGIPTYMDDFKYGFPSSGLSLQTMKW
WGTDSHTETTPAKDDNGEAPESANEASKGMTDDELDWGADEAEAEADADSAITTEPSAQLCSLRRK
AVDDGRKLLTGKSCGGLELCRLNKRQKMALAQVFGASLPEQLRSKLG
>OsRKD3 2 in 2005 (OsRKD2)
MEMHECCYYGGSIGGDWLNPLAAIPPPCSSSSSSWSSQLLLLSDHDDVLLHSAGDHGGAVAGIGGA

CMTADLVVRDEEMEMAAGYLPVAASAAAAADVDHYMYQQFQLEPDQFVSTLPAVAVAVAATAG
GGSHDDELLRMPFTDIDLDAFADARDVVVGVGEPKPSPQHTLDAAIALPAVGGGGAHHFGTQDDD
VKFDVTKQRNDAALAGDDSLSMVIVESYEMGMRRHAAEQEQEQKPKIITSAATTLTPLPLPPPPPPPP
RVTRSRRDGSSAATAGGKTRLDHIGFEDLRRYFYMPITKAAREMNVGLTVLKKRCRELGVARWPHRK
MKSLKSLILNEMGSKGMSAAAMRRELEALENCCALMERNPAVELTERTKKLRQACFKENYKRRRAAA
VDVLDLDHCFSFAAGHCHRHHHQQLALPPPPAAAADHRRRDFFGY
>OsRKD4 in 2005 (OsRKD5)
MDAAAAAVSTLSALAVFASTLDHGAVRSVHGYKVYGRGGRRRWERWVEREFVLTPASCREVPAPVA
PPRILPAEWRGRPAYREGQVVAAGAWRCILAFDSAAAPPRTPPPVLSPFLNPRLMCVPSLYNDLEKVF
RFQNVEKIPKLMQCDSEEKLSSWDARDKSSDEVHASESDSDDDLQSGEEEKPTVQKQRRANKKHIAS
ITLVDIAQYFHLPIREASRTLKIGVSILKRKCRQYNIPRWPHRKIKSLDSLIQDLEYVIDDGDDHDDTGDD
VQQEKHKQTAEEKQEAIMALTRRKQMLETEKETIQQIPAMDLKVETKQFREDVFKRRYRAKKDLAND
>OsRKD5 (OsRKD2)
MRHNRSLAALLRAGRYGAARRLFDALPARSVVTWNSLLAGLARRPDARAAREFFDAMPVRDAVSW
NTLLAAYSASPHPDHLAAARRLFDEMPQRDVVTWNTLLGAYARRGLMDEARRLFDEMPQRNAAS
WNTMVTGFFAAGQVVKALDVFDAMPAKDSASLSTMVSGFTKNGMLHEAEELLTKRLSVTDMDKAV
DAYNTLIVAYGQAGRFSDAKRLFDMIPKGQYQHNMLKRKGFERNVVSWNSMMICYIKAGDVCSAR
ALFNEMPDKDLVSWNTMISGYTQASDMKESEKLFWEMPDPDTVSWNLIIQGFMQKGEAEHARGFF
DRMPERGTISWNTMISGYEKNGNYISSVKLFSKMLEVGEIPDRHTFSSVLAACASIPMLGLGAQIHQLV
EKSFVPDTAISNALITMYSRCGALNDAEAIFKQMHTKKDLVSWNALIGCYEHHGRATKALQLFKEMRR
AKVMPTHITFVSLLSACVNAGLVSEGRMVFDTMVHEYGIVARIEHYAALVNLIGRHGQLDDALEVINS
MPMAPDRSVWGAFLGACTAKKNEPLAQMAAKELSTINPDSSAPYVLIHNLHAHEGKWGSAAVVRE
EMERQGIYKQPGYSWIDLEGKMHVFISGDTWHPNAQEIFSVLEDWQWHNPMSLEMNQLTQVKLIA
KRNNQIMDKQATATKMHKATNIRHIYSKFKVDEQQNTSWWPYCTSLWPDSYLLEEEALFSSLSFPSF
HPQPVYSTVMQSNVLQDELGVIFEDDVLKYWDEMEQSENKVEKSEKGLPLLYYGDENGAASKIMRD
DVRSEEKALTFELVSQYFYMPITQAARELNVGLTLLKKKCRELGIPRWPHRKMKSLQTLINNVQVLQEA
SKANNEEQLRMLVEMLQEERRLLEQKPYVQLEEKTKRLRQACFKANYKKRRLLALEAGEP
>OsRKD6 (OsRKD2)
MEKWRFYGGHQFQSYSFHEEENLFQDWSLDYLLLGEDEPFFTHHFSTSVHSNFVQDELYTLFDGDILS
IWGDMKEDAYHRSDKDGGEKEEKLDHEKAMELQLQRLPSGRQSGEKTLTFELVSQYFCLPIKQAAQE
LNVGLTLLKRRCRVLGIPRWPHRKVKSLETIIKNVQELGMETGQDEDNTRNAVEMLQQTKKLIEQSPD
AKLDDWTKMLRQACFKENYKRRRLLAIEG
>OsRKD7 (无)
MAMSRATCSTRPRISRCSPSRRWFVPVAAAIESHPTPAIHVVEREWPAQVQISRFDPCSPPPPARMLP
GGQQQQPHPAPGGLDLRSDEIHALKQRRRPPPNPQSSSPPEGLLGAANPDRPDDDELDAILSSFRDE
AAHGDSGGGVARVQVLPVEQDANLVPSLPLRHGQLDCSRCHLVRHVMHVAVLPYVKFSLKLVWEE
AFHRIRRMYVISVRSNVYSLSKWTQEWASEFIARNIDTMRNNTNGQLLDSGYSNLVESVRTNVNVPH
TAVEVNLLQTIMSAPSADHHQNAADQVAAPAAQPFSAAPPVALPPKAAPRKARKDRDYASMLVAV
EEFYVAATSRPVPNSDVEILESSHVSQQQDGGRAIIYPSLQARRGKTKQEVPRRNAKDVLEYLSLARKE
TEKEINTLSSFDGIYRNDGTLSYLMTEVRRLNRKIWRLQKNAPSTLSSRLLASVKEIDDIKVEKGRLYAQF
ISALKKLCRKKMDDGGSAPSANN
>OsRKD8 (OsRWP-RK2)
MAGDGGNGAADGGGEHLELDFFPSPLLDNFDVLELLDLSVLGDVDDDGAPPAPAPVQMSVDGAG
GGRINKPGSSVDDLVDWSTAFGNNCSKIDGEYGGASTSSSAAPALAPPQEDYCSGCQVLREVVHSN

GLEITKLCIHGGVASGEFYHAILDVYRVSASAPAPALAHHSIINFRGRGYDWVKQYLTEYALRRAGGGF
AVVQDSLSAFHDALCTTMAPCSSHVGDDDAHRRASSSAAAAAEERTNGNGDHGQLVVHNAAVLP
MLESSRCLVAADQAATTNNNGSGDRRLVVLDTTAIQPPASGCILHMAFPSKSYQSSRPTTLPSRYQE
CTCNPVAYEMDGVRSSLAPICWPELLGYNVTNREKTKQLQLSDIAPYFELPIAKAAKKLDICATALKGIC
RKHGVLRWPYRKVRSIDRQIATLRRSGNGDATRNEIETLIASRRRIVAGLDQ
>OsRKD9 (OsRWP-RK2)
MATGGSSSSNVPSGGAAGGGAGSGKLPMVPVGSIECLRKRCRQLRVLIHVNDHRKAVVVLHAGED
GKPDHVLVQNVSPEGEASVQSTYRIDVSGETPESQAEMLNDWYTSFRMDTTGVLYDSDQNVIYGVP
RGHPGGDVPRSLAILPPAPKKNQHGKAPATESNSSLVEEPLLLVQTDQPAAIGKRKKFTFPDQRKRVK
TMTKKDLESYFHITQKSAAHIGLSIGTTALKNLCRANDLPRWPYRQIASLDNKFNNNLKKQITGWNLG
KAVQGVTKAFKLRKEKEEFYQKIMSSMPEQLQSIDEIVNSLPEADDDIDIEDDEDNDDVIEDNDDDNS
DEN
>OsRKD10 (无)
MAGEHRFYDSNGDVSRYMLDDMEFLPLFGEAEAATTLPLPVLPDVEPLPTAPAPAATAHVEPAPAAD
FGLGNPVLADLGFDVDLDFFPELNFQSPPPPPAMNAGGYTHQVQASPPVMHHQQQQQPLAPLP
AAHGFAGQPAPATTMAPSGGDDGLFLAAPSRDAPCSPVMFNFMDFNVDMGDVDMDDVLMWA
DQDTHGAAAGGDTAPPVVVDEYADFVPFQAGDLDCSNCHLVREMMHANASRTIYFLVHATGVGS
FQHAIVDRRYTATGAEGLHFPGRQLLYFDLTNHTIESASDFIASNVEKLKNDTTGHHFLDTGYNFSGA
VRTDMANSHTAMEMNMLHTIVSAPFENVTTDAASPPAAQFIGAPPAAELPAPVPAPAPPAAHEQN
AVVATLLFKVEEFYAAANSRPAAKRADVKILESSQVTQQAGGSSAATATMYPSMVDRKRKRAQATPS
RMAPHEVIQYLRATAVETDKELETLNNFFKVCDGEDKALITFSVEQIRSIKKKIGRIINKPVTAMSSRRM
ARFIDEIDTIKEEKARVFEEIIKILKNPRRKRENDGSSGSNRKNVGGSSGGKKKTVGGSSGSKKKNVGRP
SAKKAQK
>MeNLP1
MPEQEDDKSKENQTVGGDKGETLMDLDLDLDTSWPLDQISYLSNPLSPLLLSSSDQPCSPLWAFSDA
DDDRLLPVSSASHATTPPLRFSDYPIFLACAPNSVAENHTENDDKRKFPSPLLGLMPFDNPDGYCVIKE
RMTLALRKFKESTEQHVLAQVWAPTKNGGRYVLTTSGQPFVIDPHSNGLHQYRMVSLMYMFSVDG
DCEGELGLPGRVFRQKLPEWTPNVQYYSSKEYSRRDHALHYNVQGTLALPVFEPFGQSCVGVLELIM
TSQKINYGPEVNKVCKALEAVNLRSSEILDHPSTQICNEGRRNALAEILEILTVVCETHKLALAQTWVPC
MHRSVLAFDGGLKKSCTSFDGSCNGQVCMSTTDVAFYVVDPHMWGFREACLEHHLQKGQGVAGR
AFLSHNACFCPDITQFCKTEYPLVHYARMFGLSGSFAICLRSSYTGNDNYVLEFFWPLSISDNYEQNAL
LGSLVATMKQHFQSLKVASGMDLEEEEGFVEIIQTSMSGRLDLRLECIRIPQSPKSPPNLNTSPKEGKT
AQLDPLKHHLMVNLDAVDNGGNISQAEGSHFPTSLPENKGNKKPSERKRGKTEKSISLEVLQQYFAG
SLKDAAKSLGVCPTTMKRICRQHGISRWPSRKINKVNRSLSKLKRVIESVQGAEGAFDLTPLTSPLPVTV
GSISWPSNLNGCNQQNSLNSKSLEPIDEKNGSPICNAAGNDGQGGGEAQLLGGRILSREEAVLQNG
FSPKIGLVSDRFKAGNASGEESTGTPTSHGSCQGSPANDSAPANDSPVSPVHEKCKNAGNTPELAFQ
TKGKLNLSAAYSVPDALVATEAQAPLGEMLIKDAGSSKDLRNLCPSVADTILEERVPESSWTNYPGPD
FPTTEAVAALEHAMTQGTTRQEMMTITIKAAYREDIIRFRISLSSGIVELKEEVAKRLKLEVGTFDIKYLDD
DHEWVLIACDADLQECIDISRSSGSSIIRLSVHDVNTNLGSSCESNGEL
>MeNLP2
MAEQEDDKSKDNQSVAGNKGETLMDLDLDLDTSWPLDQIYFLSNPLSPLLLSSSDQPCSPLWAFSD
ADDERLAASSSSHATHPLRLSDYPIFLTCNPSSLAESQTENDDKRKLPSPLLGLMPFDNPDGYCIIKER
MTQALRNFKESTEQHVLAQIWAPVKNGARYVLTTSGQPFVIDPHSNGLHQYRMVSLTYIFSLDGEFE
CEPGLPGRVFRQKLPEWTPNVQYYSSKEYSRRDHALHYNVQGTLALPVFEPSGQSCVGVIELIMTSQK

INYGPEVDKVCKALKAVNLKSSEILDHPSTQICNEGRKNALAEILEILTVVCETHKLALAQTWIPCMHRS
VLAFGGGLKKSCTSFDGSCNGQVCMSTTDVAFYVVDPHMWGFREASLEHHLQKGQGVTGRAFLSH
NACFCPDITQFCKTEYPLVHYARMFGLTGCFAICLRSSHTGDDEYILEFFLPPGISDVFQQKALLGSLLA
TMKQHFQSLKVASGMDLEEEEGFVEIIQTSVSGKLDLRLECIQTPESPESLPNVNTSPKEGQMAQVDP
VVNLDVVNNGGSTGDAEGGHFPTSPPENKGNKKPSERKRGKTEKTISLEVLQQYFSGSLKDAAKSLG
VCPTTMKRICRQHGISRWPSRKINKVNRSLSKLKRVIESVQGAEGTFDLTPIATSPLPVAFGSISWPSNL
NGCNQQNSPNSRSSEPNNERNGFPICKVAGSEEQSGSENQLPGGRVISHEELFLQNCFSPEIGQGSS
RHKAGNVSREESAGTPTSHGSCQGSLANDIAPAKVASASPVQEQGNEVGTIPELSFQPKGKPNLSAL
YPIPDAFVATEAQAPFRERLIEDAGSSKDLRNLCPSVADTMLEERVPEYSWTNHPGPEFPTKQSMAAL
EHATPRVTARQEMRFVTIKATYREDIIRFRISVGSGITELKEEVAKRVKLEVGSFDIKYLDDDHEWVLIAC
DADLHECIDISRLSGGNIIRLSIHDVNTNLGSSCESNGELLLGCTYGKV
>MeNLP3
MLGTRADSAMDFDCMDKLLLEGCWLETIDGSEFFNPSPFSSAAFVDASFQWPTSEINNCDLASSPSK
RSNREEEQISLLPRNSALNEAHGRSLINAEALDHGVGSADRLGYNTTEGSEVSRRWWIGPRTNPGPKT
SVRDRLIRALGYIREFTKDKDVLIQIWVPVDKGGRRVLTTHDQRFALVPNCQRLANYRDISTNYQFSVE
EDSKDMVGLPGRVFLGKVPEWTPDVQFFRSDEYPRVDHAQQYDVRGTLALPVFEQGSSTCLGVIEV
VTTKQKIKYHPELESVCMALKAVDLQSSEVPSLQHVKMCDMSYQAVLPEIHEVLRSACETHKLPLAQT
WVPCIQQGKGGCRHSDNNYYRCVSTIDLACYVHDTGVQAFHEACSEHHLLKGQGVAGEAFLTNQP
CFSSDITSYRKTEYPLSHHARMFGLHAAVAIRLRSLHSGAADFVLEFFLPAYCTDPEKQKEMLTSLSITV
QQVCQSLRVVTDKELEEETGCPISELLASSVDTPPKEEMLVTQPYSVSYGGESLSHADRLTGIQQSGSV
VSSCQKKKQKVLEDQISVDCWQNQEDYSLKVSVESGGDSAVAEGSFSSVFKGKKGEKRRTKAEKSITL
QVLQRYFAGSLKDAAKSIGVCPTTLKRICRQHGIKRWPSRKIKKVGHSLQKLQLVIDSVQGASGSFQIG
SFYTNFPELASPNLSRSNLFSTLKQSDHPEPSSIQSERVIFSSQAATPKSPSSSCSQSSNSSHCISSGTQL
HASAVTTPTSEDPVLGENSDNCVLKRARSDAELRASIQEQNLLPRSQSQKSLGEQPNLGNLLPLPNN
SSSILRDFDTQRVKVTYGNEKIRFRMPNNWELKDLLQEIARRFNIDDIHKYELKYLDDESEWVLLTCDD
DLEECIDVCQSSQTHTIKFLLQVSPHLFDRSMNSRGLS
>MeNLP4
MDHGASALNSTVWTFSSTTMDLDVMDKPLSQGYCLENNDRANSLEPEPFTSHVLYDHSEYSSFSED
LYHQIYRKEMKPLFENIPSAYHGIEGAETAKSFVQSESFTLEGSNSGSRWLIDPSYSVKQRLMLAIKYLKE
YVEDSDVLIQIWVPTQIGGQNVLTTIDQPYSLNPSCKSLASYRNVSKTFQLTAEENLKELAGLPGRVFL
GKLPEWTPDVRFFRSDECPRKSYAKMYNISGCLALPVFEQDNGVCLAVVEIVTTTQKISYSLELEIVGRA
LEAVDLKSSQDFFPLGIKACNESYQISILEISDILQFVCKTHGLPLAMTWALRDRQGEVEHWQFSKKYD
YCLSTVDSACYVADSDLLGFHEACSEHYLFPNQGIVGKAFTTKKQWFATDITSFSKANYPLSHHARMF
NLRAALAVPLCNIYTGLIEFVLELFFPWDCKDIEEQKHFWDMLSIVMQQACKSFHVIVDKEIDEEVSEQ
MVVASDGRYNREGIQELVASPSNESSQEESSSTPHEMDSQNKDKFSFISWECPKEELEDEFKVITHWE
NDEMGLYQEPVFSDFQKATQSSMPKPGVDIAENFSNGRHCSSSSEKASDKRQTKIEKTITLQILRQYF
AGSLKDAAKSIGVCPTTLKRICRQHGINRWPSRKIKKVDHSLRKLQLIVNSIHGAEGLIQIDSFYKSFPEL
SSSKFSGNDPFSSSRMSENSCILKPQPRSRLFSPKGKVPKSRSSSCSQNSGSTDEEKRLTTINGLNTGCS
LAIDDPVLMLKKAYSKPELHNLNQEEKEPKVLKSSLSHKTLHGNTTGHSLQDGMVFRVKATFGEENIR
FSFLPNWDFRDLQQEIAKRFKIDDFSRIDLKYLDIDQEPVLLTCDADLEECIDLLRLSQCNTIKISLHQAS
KSNLGSSFRIKGLF
>MeNLP5
MEDGGFKTDSVSRNLPGAAMDLDFMDELLYDGCWLTTDGLNISQPGLSSSTVPNDSSQYFPFVDTN
LHQQINQDGMENNFPENPPLSNPKVEQLADDESQDHKSVKITTSSVSAGFPNEGSELSRSFWIEPRA

DPGPTSSVKVRLMHAIRRLKECTKDREVLVQIWVPTKKEGKHVLTTTDQPCFVSLKSESLARYRYVSEN
YHFPVEGDSKESLGLPGRVFLGKLPESTPDVRFFRNDEYPRKTYAKQYNISGSLAVPVFERGTGTCLGV
VEVITTTRYINYRSELETICKALEAFDLRSSQDFCPPSVKACKEFCQAAVPEISEILGSVCETHKLPLALTW
ARCFQQGKGGCRHFDEKFANCISTVDSACFVDDRECLVFHAACSEQFLSLGQGIVGRAFVTNKQCF
ASDVTAFSKTDYPLSHHAKVLGLHAAVAIPLRSTHDGSADFVLELFLPKDCRDPEEHKQMQELLPIAIQ
QACRSLQVVMDKELEASSLNEPYPLGSSWFAQMLEAQQKGKRVCVSWDNPEEPKEEFKVATHWDE
GLEKLYEGQVFAESGQLQQNSGRKDSTEGGNNSSFAGQQSLGSRKTGEKRRTKTEKTISLEVLRQYFA
GSLKDAAKSIGVCPTTLKRICRQHGISRWPSRKLKKKTFINDE
>MeNLP6
MENLFSLKEKGKGYWTSPRAQADGMAPSDGGAKNSISEDIFNSFSEFMNFDTYAGWCTSPSATDQ
MFASYGSSSFQSTPYSTFDALSFAEQSCLTSLVGGNALNAAGTSYSSGDKMAVQQVNVYASDLMDA
DDLCAKERTGAQRQIEEMANCMISRPVGFSLDEKMLRALSLLRESADGGILAQVWVPMRRGDQYILT
TFEQPYLLDQSLAGYREVSRTYTFSAEVTPDLPLGLPGRVFISRVPEWTSSVVYYSIAEYLRGQHARNHK
VQGSIALPIFEPPDNVCCAVLELVTVKEKPNFDSEMENVRLALQAVNLRSTAPPQLLPQSLSRNQRAA
LAEITDVLRAVCHAHSLPLALTWIPCHYMEEAFDEVMKVRVREGNSRSSGKFVLCIERTACYVNDREM
QGFVHACSEHYIEEGQGIAGKALQSNHPFFFSDVKAYDITKYPLVHHARKYGLNAAVAIRLRSTYTGD
DDYILEFFLPVNMKGSSEQQLLLNNLSGTMQRICKSLRTVSDAELKGGEGSAVDFQKGAISSFPPLSV
SITSSQTTSSEAVLNLTDKVPLDASSSKYDAMKSDGPHEQSFNASRRQPEKKRSTAEKNVSLGVLQQY
FSGSLKDAAKSIGVCPTTLKRICRQHGISRWPSRKINKVNRSLKKIQTVLDSVQGVEGGLKFDPTTGGF
VAAGSIVQEFDSQRIFLSPDKNLPARNSQSATEEAVSVPSVPCIDGSNSAVKVEEDEFCMDTCGEVLM
KSSIPVIDCSEDSKSIATDAEICQKGRLGCGPWAAMDNASAFAKGTKGSLNSGSAKVDNSDTHFVSR
SSCSLGAAEVLDTKEEGDDVMVEHNQPTCSSMTESSNGSGSMIHGSASSSPSFEEKHLKVKCDDGGS
KISVKATYREDTVRFKFEPSAGCFQLYEEVAKRFKLQNGTFQLKYLDDEEEWVMLVTDSDLQECIEILDY
VGKRSVKFLVRDSVFTMGSSGSSNCFLGGSS
>MeNLP7
MEDHILSPGTMLLGAHADSTMDFDYMDKLLLEGFRLESIDGSEFFNPSSSSSAACVDSSFLWPASEIS
NGNFASSRSNQEDEHILLPRIAPPNEAHGRSLVTAQAHGEDIGSAYRLGDNTVDGSEVSRRWWIGPR
TSPGSKTSVRDRLIRALGYIKDFTKDNNILIQIWVPVDKGGRRVLTTHDQCFALVPNCQRLANYRHVSI
NYQFLAEEDSKHMVGLPGRVFLGKVPEWTPDVRFFRSDEYPRVHDAQLCDVRGTLALPVFEQGSRT
CLGVIEVVMTKQKIKYHPELERICKALEAVNLQSSEVPSLQHVKACDTSYQAVLPEIHEVLKSACETHKL
PLAQTWVPCIQQGKGGSRHSDENYYRCVSTVDHACYVRDSGVQAFQEACSEHHLLKGQGVAGEAF
LTNQPCFTSDLTSYGKTEYPLSHHARMFGLHAAVAIRLRSVHTGTADFVLEFFLPADCKDPEEQKKML
TSLSIIIQEVCQSLRVVTDKELEEENDLPISEVLALSYGTPPDEEMFRVTQPYSESYHGDNLSWSDSFPGI
QQSGSAVSSFQIEKQKLLIHEKSVDCRQTQEDYSPKVSVEYGGDSAIAEGSFSSVCVSKTGEKRRTKGE
KTITLQVLRQYFAGSLKDAAKSIGVCPTTLKRICRQHGIKRWPSRKIKKVGHSLRKLQVVIDSVQGASG
AFQIDSFYTKFPELASPNLSRSTPLSTSKQLGHPESSSIQPEGGIFSSQVAAPKSPSSSSSQSSSSSHCCS
SGTEQHASTLTIPTCKDLMLGENSGNCVLKGVKSDAELHVSIQEEQNLLPRSQSQKSLWEQPNLGNL
PPLAKSSNRICQEIDGQRVKVTYGNEKIRFRMPNNWELKDLLQEIARRFNIDDIHKYDLKYLDDDSEW
VLLTCDDDLEECIDLCQSSQSHTIKLLLQISPHLLDRSTNSRGLS
>MeRKD1
TKDNERKRSAPLELEEIRKHFDVPITKEAKEMKVGLTILKKRSRELKIMRWPHRKIRSLKSLINNIKEMGLT
NEIMMLEEHQRLLEKKPDMEFSETTKKLRQAIFKANYKKKRCLVAHHI
>MeRKD2
MDFSQDYSLTALRIFQNTINREFIRSLHVYRLKDGTDKEVEREFLFSDDGPYVEMVANPLLRLDRFRVLE

LFEGQVIGVWHCIFAFNAHHSPHLSRIPSLLSISRNPKLKSVPTLANDLQLIFKLISRTADEEPLQFLSEEK
CRMIKGCFQSKRNLPVLDQDLNCLPYSVATSQVPKSQQIEPSEPASVMAKKKKRAATEDIARIALEDL
VKYFDLPIAEASRNLKVGLTVLKRKCREFGIPRWPHRKIKSLDSLIRNLQEEAERQKQENEDAAMAVAK
RQKMLEREKESIERKPFMEIQSETKRFRQDVFKRRHRARALKTQGLRVSQASA
>MeRKD3
MDSKITVELMNFPKNDTDCFLMDEYFERLSELSPLESFLEFDSLSLPYDTNFGFEDYLLENGHPLSWDV
DIPIDAKPLRVFTSTIDNTVKYDDDFTGTSSNEEEKRVIMGRKRSAPLEMEEIRKHFDKPITKAAKEMKV
GLTVLKKRCRELSIMRWPHRKIKSLKSLIRNVKEKGLTNEAVMLEEHQRLLEKMPDMELNDSTKKLRQ
AIFKDNYKKRRCLEAHA
>MeRKD4
MADPRYVVPYHDPHDSPINYEILNFLRDANPTLENLPSTDEPTSLHVSSDDIVNPNLSLEDPMIWDIV
NQPNSGGQSQGEGPSLNRERRATENLEQRYLDDGKPISVWPPPAMPFQCTCCQVLREIIHTDGNCT
TKLEIHGRLGIICHAVLEIKDQVMYEAQQADQTEGGNEAERGSRSTLAQQRERTGRLTLKDFEAYFHL
PIEEAAKIMNLCPTVVKKICRRYGMTRWPHRKIKSIQRQISNLRANSNWNDPGERARAQAEIQRLEVEI
SNICSGVTN
>MeRKD5
MGLTLLKKRCRELGIRRWPHRKLMSLQTLIKNVQEMKKVEGKESEEKLREAIEILESERKMLEEIPDLQLE
HKTKRLRQACFKSNYKKRKLMAMMSRPSSSSGAIDYYGQINEDEDEEIKSLLADPVCHPTMMF
>MeRKD6
MANWQNELATQESFLAAYPLIESFVPDPFCASLEIENSTIHGDQDVNGLGDWNEFSTLFDPQKQLLL
SCDSARGSSRTEPFEEEKKAKKCREEKVNNSISKALSRQTISMYFYMPITQAAKELNVGLTLLKKRCREL
GIRRWPHRKLMSLQTLIKNVQEMKKVEGEESEKKLREAIEILERERKMVEETPDMQLENKTKRLRQACF
KANYKKRRLMGMIDKPSSSSSSTITNKMMNEDEENIEEYEEIISLLADPTSHTNMLF
